# A solitary fibrous tumor of the parotid gland: Case report

**DOI:** 10.1016/j.ijscr.2023.108855

**Published:** 2023-09-22

**Authors:** Carmelo Saraniti, Davide Burrascano, Barbara Verro, Giovanni De Lisi, Vito Rodolico

**Affiliations:** a Division of Otolaryngology, Department of Biomedicine, Neuroscience and Advanced Diagnostic, University of Palermo, 90127 Palermo, Italy; b Pathology Unit, Department of Health promotion Sciences maternal and Infantile Care, Internal Medicine and Medical Specialties, University of Palermo, 90127 Palermo, Italy

**Keywords:** Solitary fibrous tumor, Salivary gland, Parotid gland, Histology, Differential diagnosis, Multidisciplinary team

## Abstract

**Introduction:**

Solitary fibrous tumor is a rare neoplasm that can affect any part of the body, also head and neck region. Etiology is unknown. The incidence is slightly higher in males, the age ranges from 11 to 79 years.

**Presentation of case:**

It's the first case in our country of left parotid solitary fibrous tumor, removed by partial parotidectomy with facial nerve preservation. Histology examination showed diffuse spindle-shaped cells proliferation, moderate polymorphism, low mitotic index (<4 mitoses per 10 HPF), partially bordered by fibrous capsule. Immunohistochemistry showed STAT6, CD34, CD99 positivity. Six-months follow-up didn't show sign of recurrence.

**Discussion:**

Solitary fibrous tumor is a mesenchymal spindle cell neoplasm with fibroblastic differentiation ubiquitous in soft tissues, that involved the head and neck region in 6 % of cases. Etiology is unknown. The possible pathogenesis is NAB2-STAT6 gene fusion. It's asymptomatic or symptoms are related to space-occupying mass. Diagnostic work up involves imaging, immunohistochemistry, histology. Radiographic finding may lead to incorrect assessment of the mass: the same imaging features are present in pleomorphic adenoma, the most frequent tumor of salivary glands.

**Conclusion:**

This case report aims to stress that, although rare, solitary fibrous tumor should be considered in differential diagnosis in case of indolent salivary gland mass, since it may require more invasive approach (e.g., total parotidectomy, adjuvant radiotherapy). It would like to highlight the role of multidisciplinary team to define the best therapy, tailored for the patient, as well as to give awareness to a rare but sometimes aggressive tumor.

## Highlights

• Solitary fibrous tumor (SFT) is a rare tumor.
• The diagnosis cannot disregard imaging techniques, immunohistochemistry, histology.
• Parotid SFT is in differential diagnosis with pleomorphic adenoma.
• Therapy depends on histological data with a multidisciplinary approach.

## 1 Introduction

Solitary fibrous tumor (SFT) is a mesenchymal spindle cell neoplasm with fibroblastic differentiation that usually develops in the pleura but can also affects extrapleural sites: 6 % of cases involves the head-neck region [1]. In particular, in case of salivary gland involvement, SFT is described as a slow-growing, solid mass, generally asymptomatic. Less frequently, it leads to non-specific symptoms, including facial paralysis and, when it reaches significant size, respiratory distress [2]. The etiology is unknown, although the possible pathogenesis lies in the NAB2-STAT6 gene fusion [3,4]. Statistically, the incidence is slightly higher in the male sex (1.2:1 F:M ratio) and the age ranges from 11 to 79 years [5].

Our case involves a female patient in her 20s with a mass in the parotid region suspicious for pleomorphic adenoma that comes at our Ear Nose & Throat Unit in University Hospital. This work was written according to the SCARE criteria [6].

## 2 Case report

A 20-years old Caucasian woman presented at our ENT unit with a visible neoformation in the left parotid region for 2 months. Her past medical and surgical history were not significant, but her family history was positive for cancer: her mother underwent surgery due to breast cancer, and her father died due to a liver cancer.

Physical examination revealed a 3-cm oval, semi tense-elastic mass adhering to the deep planes, painless and covered by intact skin, in the left parotid region. There was no sign of facial paralysis. Her blood tests, that were White and Red Blood Cell, Hemoglobin, Hematocrit and liver and kidney indices, were normal.

Ultrasound of the neck showed a voluminous eco-solid nodular formation with regular margins and some internal areas of colliquation, at the lower pole of the left parotid gland. Some increased reactive-inflammatory bilateral neck nodes were also found.

**Fig. 1:**
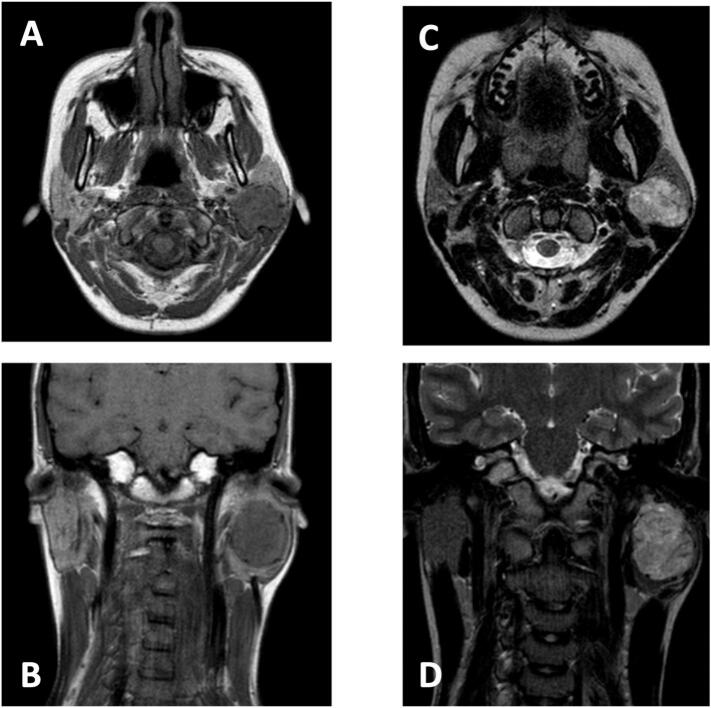
Magnetic Resonance Imaging showed an oval-shaped neoformation, medium-low intensity at T1 on axial (A) and coronal (B) scans, high intensity at T2 on axial (C) and coronal (D) scans.

Magnetic Resonance Imaging (MRI) showed an oval-shaped neoformation, with lobulated margins, medium-low intensity at T1, high intensity at T2 and STIR and remarkable and homogeneous enhancement with medium contrast (Fig. 1).

Patient refused fine needle aspiration cytology (FNAC) because she was afraid of the procedure, so it was not carried out.

Based on the anatomical site, the higher frequency of incidence, the clinical and radiological features, the first suspected diagnosis was pleomorphic adenoma. So, the therapy was superficial parotidectomy.

The patient underwent left superficial parotidectomy under general anesthesia. A skin incision was made according to Radon and the SMAS (Superficial MusculoAponeurotic System) flap was elevated. The posterior margin of the parotid gland was highlighted and then cleaved from the sternocleidomastoid muscle. The digastric muscle was detected, and the facial nerve was identified. Its integrity was monitored and checked during surgery by Nerve Integrity Monitoring (NIM) System. So, the lesion was excised with the superficial portion of the parotid gland preserving the integrity of the facial nerve.

Histological examination reported a randomly arranged spindled cell proliferation composed by cells with mild nuclear atypia, pale eosinophilic cytoplasm, within a variable collagenous stroma admixed with branching and hyalinized staghorn-shaped blood vessels. Signs of necrosis, angio-invasiveness and perineural infiltration weren't found. Mitotic index was low (<4 mitoses per 10 HPF).

The neoplasia was partially bordered by fibrous capsule and showed expansive margins towards the contiguous glandular parenchyma.

**Fig. 2:**
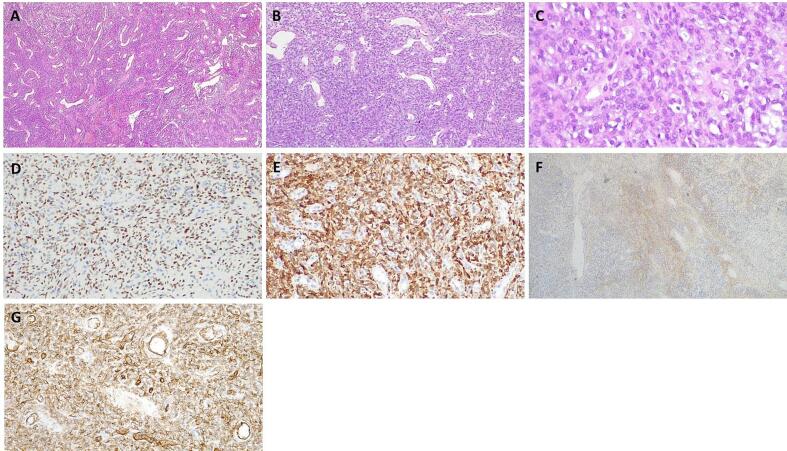
Immunohistochemical analysis: (A) Hematoxylin-Eosin original magnification 40×, (B) Hematoxylin-Eosin original magnification 100×, (C) Hematoxylin-Eosin original magnification 400×, (D) STAT6 original magnification 200×, (E) BCL2 original magnification 200×, (F) EMA original magnification 40×, (G) CD34 original magnification 200×.

Immunohistochemical analysis found that the neoplastic cells were positive for STAT6, CD34, CD99, BCL2, PAX8, Calponin, EMA (focal positivity) and negative for CD117, DOG1, 1A4 actin, S100, SOX10, beta-catenin, myogenin (Fig. 2).

Therefore, the morphological and immunohistochemical scenario showed a fibroblastic proliferation with the characteristics of the solitary fibrous tumor (*sec*. WHO Classification of Tumors, Soft Tissue and bones tumors 5th Edition [7]) with free resection margins.

In view of the mild aggressivity of the tumor, the affected anatomical site, and the patient's young age, the multidisciplinary team – consisting of otolaryngologist, oncologist, plastic surgeon, pathologist, radiologist and radiation oncologist – planned a close follow-up, with clinical and radiological (MRI, positron emission tomography – PET) examinations. At 6-months follow-up, no signs of recurrence were found.

## 3 Discussion

In comparison to tumors of head and neck region, parotid gland tumors are characterized by histologic heterogeneity [8,9]. Epidemiologically, parotid gland tumors are relatively rare: in fact, they represent 3 % of all head and neck cancers with 1–3 cases/year per 100.000 population [10]. The 2017 WHO classification of salivary gland tumors includes 11 benign and 20 malignant tumors [11]. Among benign lesions, the most frequent are pleomorphic adenoma (80 % of cases), Whartin's tumor (10 % of cases), and monomorphic adenomas (10 % of cases). On the other hand, malignant tumors include mucoepidermoid carcinoma (30 % of cases), adenoid cystic carcinoma (25 % of cases), carcinoma ex pleomorphic adenoma (15 %), and acinar cell carcinoma (5–10 % of cases) [12]. Females are most affected by benign forms, in contrast, males by malignant ones [13].

Solitary fibrous tumor (SFT) is a mesenchymal spindle cell neoplasm with fibroblastic differentiation ubiquitous in soft tissues, that may occur in any anatomic site, although it is most common in the pleura [14]. The head and neck region is involved by SFT in only 6 % of cases [1] and the occurrence at the level of the major salivary glands is very rare. The parotid is the most involved salivary gland while the sublingual is the least affected [15].

Although the etiology is unknown, the possible pathogenesis is NAB2-STAT6 gene fusion [3,4]. Macroscopically, SFT is described as a lobulated or nodular greyish slowly growing mass, well-circumscribed, encapsulated or not totally [2]. Generally, it is asymptomatic or results in symptoms related to the space-occupying mass, such as facial nerve compression with facial paralysis [16] or respiratory distress when the mass deepens to the parapharyngeal space [17].

In our case, as in almost all cases, the injury was totally painless. For this reason, progressively greater swelling is the only real warning sign that leads the patient to seek medical attention.

Currently, only thirty-seven cases of SFT in the parotid gland have been reported in the literature [5]. The current literature revealed that, both sexes are affected with a mild prevalence for the male one (1.2:1 F:M ratio), age ranges from 11 to 79 years (mean age 49.5), the most frequent site of occurrence is the superficial lobe of the left parotid gland and the least is the deep lobe of the right gland; the size varies from 1 to 12 cm [18]. The largest mass (16 × 18 cm) was described by Chis et al.: it was considered a giant SFT [19]. Referring to the literature, our case is consistent with the literature: it is represented by a female patient, 20 years old, with a 3 × 2 cm mass interesting the superficial lobe of the left parotid gland.

In accordance with WHO criteria, increased mitotic index (>4 mitoses/10 HPF), cytologic atypia, tumor necrosis, and infiltrated margins characterize the malignancy of the neoplasm [20]. Three cases of malignant parotid STFs were reported in literature, none of which had necrosis [[21], [22], [23]]. It has also been described a case of aggressive STF with destruction of the cortical bone of the mandible, even characterized by absence of necrosis and intralesional hemorrhage but with increased mitotic rate and Ki67 [24]. Luckily for the patient, our case presented features of benignity: no necrosis, angio-invasiveness and perineural infiltration, and low mitotic index (<4 mitoses per 10 HPF). These features led the multidisciplinary team to decide for a close follow-up.

The diagnostic work up involves computed tomography (CT) and/or MRI, immunohistochemistry, and histology. SFT appears on CT as a well-circumscribed hypointense mass that takes heterogeneous enhancement after contrast medium [25]. On MRI, the tumor is showed as an isointense mass on T1-weighted images and hyperintense on T2-weighted images [26], as in our case. This radiographic finding may lead to an incorrect assessment of the mass. In fact, the same imaging features are present in Pleomorphic Adenoma, the most frequent tumor of the salivary glands. On MRI, Pleomorphic Adenoma is represented as a well-defined mass with lobulated contours. On T2-weighted images, it appears hyperintense with a hypointense contour representing the capsule. The intratumoral signal varies in response to different cellular density [27].

The histologic features of SFT are variable and include a proliferation of cells that may be ovoid or spindle-shaped, clustered in a fascicular or “patternless” pattern, along with collagen deposition and a rich vascular network [28].

Immunohistochemical investigations play a crucial role in confirming the diagnosis of SFT because STAT6 has been found to be the most sensitive and specific marker [29], even if a significant subset of these tumors shows a paracentric inversion involving chromosome 12q, resulting in NAB2-STAT6 gene fusion [3]. STAT6 expression may be lost in dedifferentiated SFTs [30].

Other less specific markers usually positive in SFTs are CD34, CD99, BCL-2, vimentin [28].

The negativity for S-100, SOX10, cytokeratin, desmin and CD117 are useful in the differential diagnosis with epithelial and mesenchymal neoplasms showing the same morphological features. In fact, in contrast to SFT, neurofibroma and schwannoma are positive for S-100, dermatofibrosarcoma protuberans is often bcl-2 negative, melanoma is positive for HMB45, S-100 and Melan-A and meningioma is SOX10 and S100 positive [18]. These data confirmed and emphasized that the key role of immunohistochemical examination for proper management of the disease.

The treatment of choice is surgical excision, paying attention to possible intraoperative complications such as bleeding. In fact, when the STF is highly vascularized [25], preoperative embolization may be performed. Complete resection is the most important factor for clinical outcome [31,32].

## 4 Conclusion

SFT is a rare tumor, especially considering extrapleural sites, but reaching a correct and timely diagnosis is crucial. Suspicion should be raised for all solid, slow growing, more frequently indolent masses. The diagnosis cannot disregard imaging techniques (CT and/or MRI), immunohistochemistry and histology. The treatment of choice is surgery and close follow-up, with the involvement of a multidisciplinary team in relation to the chances of future recurrence of the mass.

The main take home messages are the importance of the differential diagnosis between this rare disease – as proved by the poor literature – and the more frequent parotid gland disease (e.g., pleomorphic adenoma) and the key role of a multidisciplinary team that ensures the best and proper therapeutic approach for the patients, most of all in case of rare tumors, like the solitary fibrous tumor of the parotid gland.

## Funding

This research did not receive any specific grant from funding agencies in the public, commercial, or not-for-profit sectors.

## Ethical approval

We acquired the consent for publication from patient but we don't require ethical approval since it is a anonymous case report.

## Consent

Written informed consent was obtained from the patient for publication of this case report.

## CRediT authorship contribution statement

Carmelo Saraniti: concept of design, writing the paper, validation of final version.

Barbara Verro, Davide Burrascano, Giovanni De Lisi: data collection and analysis, writing the paper.

Vito Rodolico: validation of the final version.

## Guarantor

Carmelo Saraniti.

## Declaration of competing interest

The authors declare no conflict of interest.
